# The Mouse IAPE Endogenous Retrovirus Can Infect Cells through Any of the Five GPI-Anchored EphrinA Proteins

**DOI:** 10.1371/journal.ppat.1002309

**Published:** 2011-10-20

**Authors:** Marie Dewannieux, Cécile Vernochet, David Ribet, Birke Bartosch, François-Loïc Cosset, Thierry Heidmann

**Affiliations:** 1 CNRS UMR 8122, Institut Gustave Roussy, Villejuif, France; 2 Université Paris-Sud, Orsay, France; 3 Université de Lyon, UCB-Lyon1, IFR128, Lyon, France; 4 INSERM, U758, Lyon, France; 5 Ecole Normale Supérieure de Lyon, Lyon, France; Fred Hutchinson Cancer Research Center, United States of America

## Abstract

The IAPE (Intracisternal A-type Particles elements with an Envelope) family of murine endogenous retroelements is present at more than 200 copies in the mouse genome. We had previously identified a single copy that proved to be fully functional, i.e. which can generate viral particles budding out of the cell and infectious on a series of cells, including human cells. We also showed that IAPE are the progenitors of the highly reiterated IAP elements. The latter are now strictly intracellular retrotransposons, due to the loss of the envelope gene and re-localisation of the associated particles in the course of evolution. In the present study we searched for the cellular receptor of the IAPE elements, by using a lentiviral human cDNA library and a pseudotype assay on transduced cells. We identified Ephrin A4, a GPI-anchored molecule involved in several developmental processes, as a receptor for the IAPE pseudotypes. We also found that the other 4 members of the Ephrin A family –but not those of the closely related Ephrin B family- were also able to mediate IAPE cell entry, thus significantly increasing the amount of possible cell types susceptible to IAPE infection. We show that these include mouse germline cells, as illustrated by immunohistochemistry experiments, consistent with IAPE genomic amplification by successive re-infection. We propose that the uncovered properties of the identified receptors played a role in the accumulation of IAPE elements in the mouse genome, and in the survival of a functional copy.

## Author Summary

In mammals, nearly half the genome is composed of reiterated scattered sequences. Some of them, called endogenous retroviruses, have a structure similar to that observed for the integrated form of infectious retroviruses. The current theory to account for their presence is that an infectious retrovirus once infected the germline of its host. This viral genome was then transmitted to the progeny and expressed from there, producing new infectious particles, which could re-infect new germline cells and thus increase the viral genomic copy number. However no evidence has yet been provided to support this model. In this study, we identify a family of five cellular proteins, the Ephrin As, as receptors for a model mouse family of endogenous retroviruses, the IAPE elements. We analyse their expression pattern and show that both the oocytes and some male germline cells express Ephrin A proteins and can thus be infected by IAPE particles. This finding strongly supports the current model of ERVs amplification. In addition, the IAPE envelope ability to use five different cellular receptors suggests that it might be impossible for the host to evolve a resistance against this viral element, and provides a clue on how the IAPE family survived so long in the mouse genome.

## Introduction

Mammalian genomes are filled with numerous copies of mobile genetic elements. Among them, endogenous retroviruses are the remnants of infectious retroviruses that once infected the germline of their host and have since then been transmitted from one generation to the other following a mendelian pattern (reviewed in refs. [1]–[3]). After this initial insertion within the genome, some of these elements were recruited by their host and some of their open reading frames changed into “ordinary” cellular genes which now fulfil physiological functions, like the syncytin genes that are involved in the formation of the placenta [4]–[8]. However, the bulk of these elements still behave like transposons and increase their copy number after the initial invasion of the germline. While doing so, they can cause insertional mutagenesis, either by directly interrupting open reading frames or by inducing dysregulations of cellular genes (reviewed in [3]).

The amplification is thought to have initially proceeded via successive re-infections of the germline using a traditional extracellular infection route. However, the most successful families of elements (with regards to their copy number) identified so far have switched to a strictly intracellular amplification mechanism that does not require the viral particles to be exposed to the extracellular compartment and that makes them much more efficient (reviewed in [9], [10]). This switch in the amplification strategy is usually correlated to the loss of the envelope (env) gene that encodes the membrane glycoprotein responsible for the binding of the particle to a cellular protein used as a receptor, and a modification of the intracellular trafficking of the particles via an alteration of the N-terminal part of the structural Gag protein. These changes can be seen in pan-mammalian ERV-L elements, as well as in the mouse MusD and IAP (Intracisternal A-type Particles) elements [10]–[12], and lead to an intracellularisation of the elements. During the process, the latter increase their amplification efficiency within their current host but completely loose their autonomy, being unable of re-infection and direct horizontal transfer, and thus cannot colonise new species anymore. The mouse IAPE family is particularly interesting in this respect: we previously demonstrated that it is the progenitor of the intracellular IAP elements [10], which are probably the most successful and active family of retrotransposons in the mouse, being responsible for an estimated 10% of the de novo mutations occurring in laboratory animals. But at the same time IAPEs also survived as infectious elements, with an identified mouse endogenous proviral copy being able to produce fully functional particles that can re-infect a variety of cells from different species. The IAPE family is for this reason quite special, since the progenitors of the other widespread intracellularised elements have disappeared from the genome of their host. We wondered whether this specificity could be due to a particular tropism of the particles, and tested this hypothesis by searching for the IAPE cellular receptor.

## Results

### Screening of a lentiviral cDNA library

**Figure 1:**
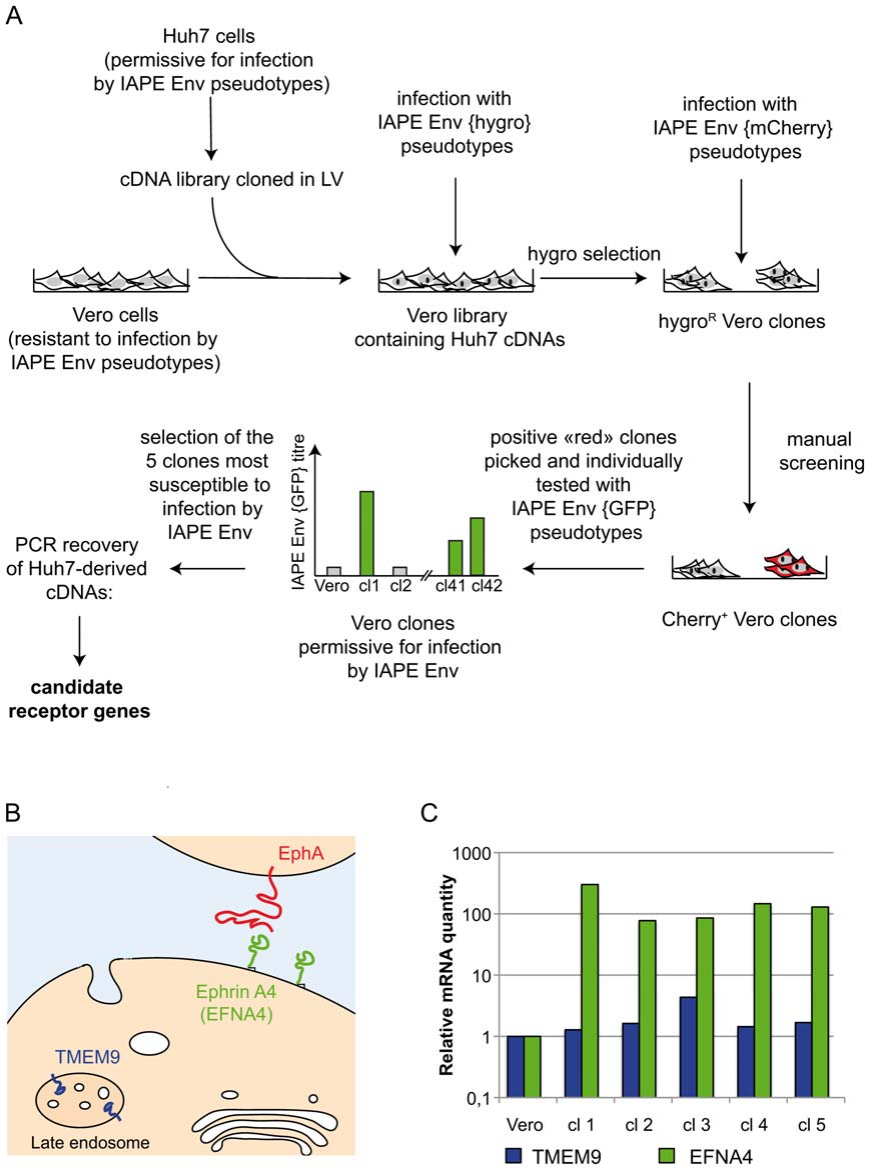
Screening strategy and identification of cDNA clones for the IAPE receptor. A. Scheme of the screening strategy to identify the protein used as a receptor by the IAPE Env. More technical details are provided in the main text and in the Methods section. LV: lentiviral vector. B. Scheme representing the cellular localisation of the proteins encoded by the two candidate receptor genes. TMEM9 is an endosomal protein, whereas Ephrin A4 (encoded by the EFNA4 gene) is expressed at the cell membrane where it can interact with extracellular molecules, including the EphA proteins. C. Quantification of the mRNA level of the two genes identified as potential receptors for the IAPE elements. The amount of the corresponding mRNAs was measured by qRT-PCR performed on total RNA extracted from the parental Vero cells as a control, or the five clones found to be the most sensitive to infection by IAPE pseudotypes (re-numbered from 1 to 5 in this panel). Clones 1,4 and 5 contained an EFNA4 cDNA, and clones number 2 and 3 a TMEM9 cDNA. The mRNA levels were normalised using the RPLO gene as a reference, and the parental Vero cell line level was set at 1 for both genes.

To identify the protein used as a receptor by the IAPE endogenous retrovirus, we made use of a lentiviral cDNA library generated from human Huh7 cells (see Methods). It was selected because Huh7 cells can be infected by retroviral pseudotypes carrying the IAPE Env (data not shown) and are therefore certain to express the IAPE envelope receptor. As schematised in Figure 1A, these cDNA were introduced into simian Vero cells, which are resistant to infection by the IAPE Env pseudotypes, using VSV-G lentiviral pseudotypes at a multiplicity of infection (MOI) of approximately 6. The cells were grown for 3 days to allow expression of the transduced cDNAs before being submitted to two cycles of infection by IAPE Env pseudotypes containing a hygromycin resistance gene (final MOI estimated to be more than 1), or by control pseudotypes containing the hygromycin resistance gene (hygroR) but with no envelope proteins. After three days of amplification, the cells were submitted to hygromycin selection. Because of the high background due to the infection protocol used with the pseudotypes (high MOI and spinoculation), we obtained a high number of hygroR clones, with no clear difference between the IAPE Env and no Env pseudotype-infected cell populations. This was not surprising since the theoretic frequency of cells transduced by a given cDNA (approx. 1 out of 10,000 cells, assuming all genes are equally expressed) is much lower that the background level of infection (1 cell out of 100–1000). The clones were thus subjected to a second round of selection using IAPE Env pseudotypes encoding the red fluorescent protein mCherry that we applied directly on the original plates (Figure 1A). The plates were then manually screened for red fluorescence to identify clones containing multiple (>10) independent infection foci. 42 such clones were identified, grown individually and then assayed a third time for their susceptibility to infection with IAPE Env using GFP-containing pseudotypes. The 5 more infectable ones (IAPE Env titre increased by 10–100 fold as compared to the parental Vero cell line) were selected for further analysis of their Huh7-derived cDNA content.

### Ephrin A4 is a receptor for the IAPE envelope protein

To characterise the cDNA present in the selected clones, we extracted their genomic DNA and total RNA and subjected them to PCR/RT-PCR using primers located within the lentiviral vector and surrounding the cDNA cloning sites. These PCR analyses showed that all the selected clones contained more than one cDNA, which was expected due to the high MOI used during the cDNA library transduction. In addition, the 5 clones can be split in two groups with regards to their cDNAs content, indicating that these 5 clones originated from only two initial transduction events. This is possible since the cells were amplified before they were infected with the IAPE Env pseudotypes. Most of the cDNAs we identified were not considered as potential receptors for the IAPE Env, either because they were truncated at their 5′ end and did not contain a complete ORF, or because they corresponded to soluble intracellular proteins. However, two of the clones contained a full-length cDNA of TMEM9 (the gene for Transmembrane Protein 9) (clones number 2 and 3 in Figure 1B), while the last three (number 1, 4 and 5) had been transduced with a cDNA of EFNA4 (the gene for Ephrin A4), both of which encode membrane-associated proteins.

TMEM9 was the less likely candidate, as it is described as a strictly intracellular protein, associated with the endosomal membranes [13] (see Figure 1B). In addition, qRT-PCR experiments indicated that it is already moderately expressed in the parental Vero cells and that its expression was only increased by approximately 4 times in one of the two clones containing its cDNA, with its level being mostly unchanged in the four other positive clones (Figure 1C). Its ectopic expression in Vero cells, that we achieved either by transfection or by lentiviral transduction, did not render the cells susceptible to infection by the IAPE Env pseudotypes (not shown), definitively demonstrating that TMEM9 is not a receptor for the IAPE Env.

**Figure 2:**
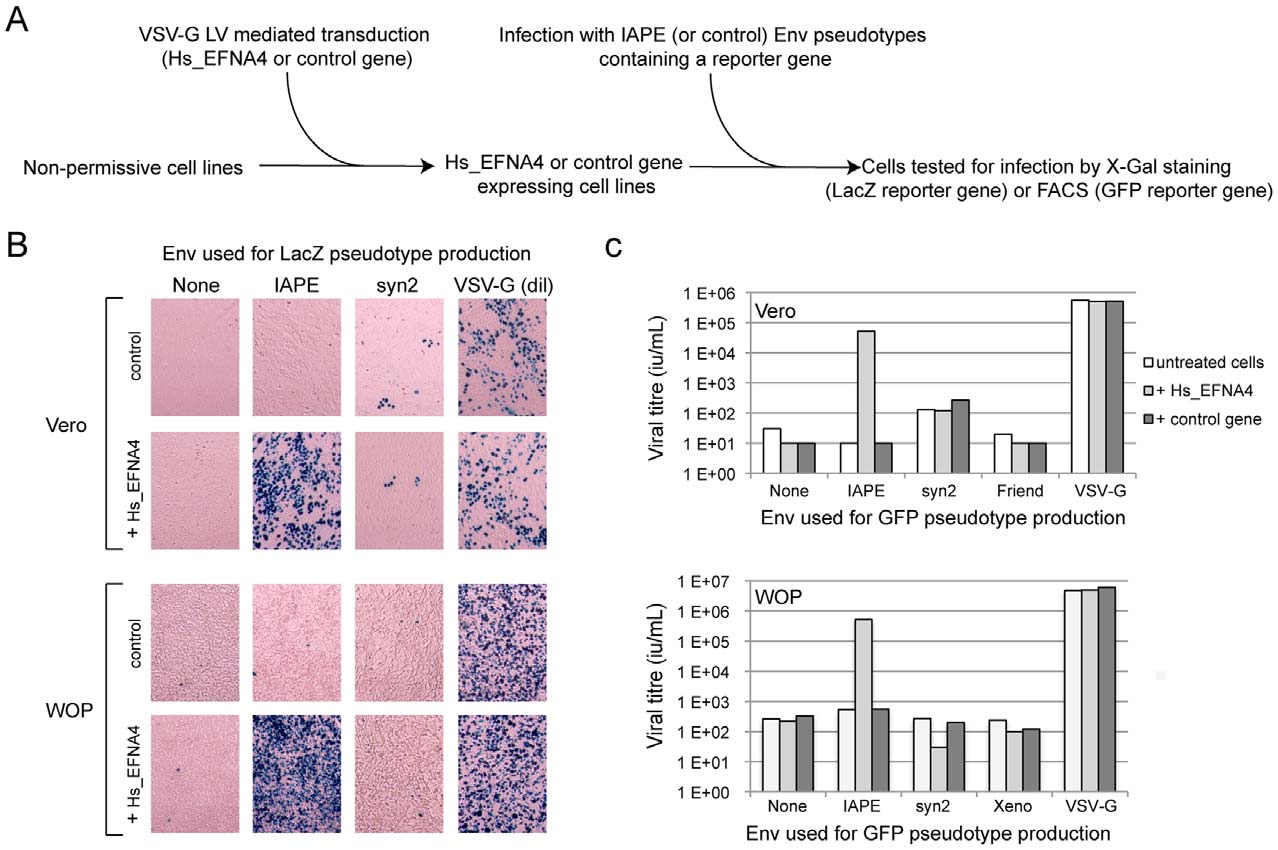
Expression of EFNA4 is sufficient to render cells susceptible to infection by IAPE Env pseudotypes. Following the screening strategy presented in Figure 1, EFNA4 was identified as a potential receptor for the IAPE Env. (A) To confirm this hypothesis, its cDNA (Hs_EFNA4 for Homo sapiens EFNA4) was re-cloned in a lentiviral vector (LV) and introduced into non-permissive Vero and WOP cells that were then subjected to infection with IAPE Env pseudotypes containing either the GFP or lacZ genes. (B) For the lacZ-containing pseudotypes, the cells were fixed and stained with X-Gal to reveal ß-galactosidase activity 3 days post infection. A photo of one representative field for each condition is presented. Note than in the case of the VSV-G pseudotypes, the supernatant was diluted 200 fold before its use for infection. (C) For the GFP-containing pseudotypes, the target cells were collected 3 days post infection and subjected to FACS analysis in order to quantify the proportion of GFP-positive cells, allowing precise calculation of the viral titres (see Methods for details). The results presented in B and C correspond to one representative experiment out of 3.

The other putative receptor we identified, Ephrin A4, was a much better candidate. As with the other members of the ephrin A family, it is a plasma membrane protein, attached by a GlycosylPhosphatidylInositol (GPI) anchor, that can interact with a set of integral membrane proteins called EphA (Ephrin A receptor proteins) (Figure 1B). In vivo, these interactions are widely used in developmental processes and axonal guidance (reviewed in [14], [15]). By qRT-PCR, we found that this gene is poorly (if at all) expressed in the Vero cell line and its transcript level is increased by 50–500 fold in the five clones we had identified as positive for IAPE Env infection (Figure 1C). Using specific primers in a PCR reaction performed on genomic DNA, we checked that only three of these clones contained an EFNA4 cDNA, indicating that the other two (that possess the TMEM9 cDNA) had overexpressed their endogenous copy via an unknown mechanism. All these findings made it a promising candidate as an IAPE Env receptor. To test it directly, we overexpressed this gene in Vero and WOP (a SV40-transformed murine fibroblast cell line) cells, and tested if it made them more susceptible to infection by IAPE Env-bearing pseudotypes containing either the GFP or the LacZ reporter gene (see scheme in Figure 2A). As shown in Figure 2B and C, these two cell lines are naturally resistant to infection by the IAPE Env pseudotypes. However, the same cells expressing Ephrin A4 (via a lentiviral vector) can be very efficiently infected by the IAPE Env pseudotypes. This effect is specific, since it did not make the Vero cells susceptible to infection by the Friend ecotropic Env pseudotypes, nor the WOP cells infectable by the syncytin-2 (syn2) or xenotropic MLV (Xeno) Env pseudotypes. In these two cell lines, the titre of VSV-G pseudotypes was not modified, indicating that the gain in the IAPE Env titre is not due to a general increase in the susceptibility of these cell lines to lentiviral pseudotypes. This set of experiments indicated that the EFNA4 cDNA we identified in the Vero clones is responsible for their acquired susceptibility to IAPE Env pseudotypes.

**Figure 3:**
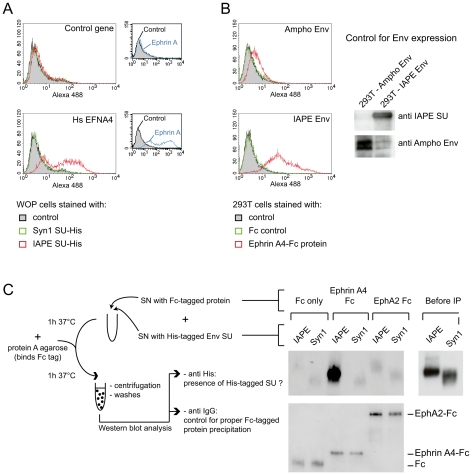
Ephrin A4 is a bona fide receptor for the IAPE Env. (A) WOP cells transduced with the EFNA4 gene or a control gene were stained with the soluble His-tagged IAPE envelope SU subunit (or that of syncytin1 (syn1) as a control) followed by an Alexa488 anti-His antibody and subjected to FACS analysis. Only the cells transduced with EFNA4 bind the IAPE SU protein, and none of the cells were stained with the control syncytin1-SU. The expression level of Ephrin A proteins in the two populations was checked using the EphA2-Fc soluble protein (that can bind all Ephrin A proteins) and are shown in the small panels on the right. The data presented correspond to one representative experiment out of three. (B) 293T cells were transiently transfected with an expression vector for IAPE Env, or Ampho MLV Env as a control. At day 2 post transfection, cells were stained with a soluble Ephrin A4-Fc fusion protein, or a control Fc protein, followed by an Alexa 488 fluorescent anti-Fc antibody before being subjected to FACS analysis. Only the cells expressing the IAPE Env bind to the recombinant Ephrin A4 protein, The data presented correspond to one representative experiment out of three. Expression of the two envelope proteins in the transfected cells was checked by Western blot using specific antibodies, as shown on the right. (C) The soluble recombinant IAPE or syn1 His-tagged SU proteins were tested for interaction with Ephrin A4-Fc (or Fc-only and EphA2 controls) in a pull down assay as schematised on the left. Pellets were analysed by Western blot using an antibody directed against the His tag (upper part). The only interaction detected is between Ephrin A4-Fc and IAPE SU. We ensured that the 2 His-tagged SU proteins were produced in similar amount, as shown on the right (Before IP panel) and that all 3 Fc-tagged proteins were efficiently pulled down by the protein A-agarose beads and recovered in similar amount in the pellet, as shown on the lower panel (Western blot performed with an anti mouse IgG antibody).

We then constructed an expression vector for a His-tagged soluble IAPE Env SU subunit, as previous studies with other retroviral envelopes had shown that such constructs can be used to stain cells expressing the cognate receptors [16], [17]. In FACS experiments, the IAPE soluble SU protein could only label WOP cells that had been previously transduced with an expression vector for Ephrin A4 (Figure 3A), whereas an irrelevant His-tagged SU protein (derived from the syncytin1 envelope protein) did not give any staining, consistent with these cells not expressing a functional receptor for syncytin1. The specific staining observed with the IAPE soluble SU protein indicates that the acquired infectability of the Ephrin A4-expressing WOP cells by the IAPE pseudotypes is linked to the ability of these cells to bind the IAPE envelope protein. However this did not rule out that Ephrin A4 could act as an intermediate, inducing the expression (or potentially altering the subcellular localisation) of another protein that would be the “true” receptor. We thus set up the reverse experiment: 293T cells were transiently transfected by an expression vector for either the IAPE Env, or a control Env. Two days post transfection, these cells were stained using a soluble, Fc-tagged Ephrin A4 protein (Ephrin A4-Fc) or a control Fc protein, and a fluorescent secondary antibody directed against the Fc domain. The Ephrin A4-Fc fusion protein has been successfully used to label the natural ligands of Ephrin A4 [18], and we used it to test if it would also bind the IAPE Env. As shown in Figure 3B, we could detect by FACS analysis a strong staining with the Ephrin A4-Fc protein in the IAPE Env expressing cells, whereas we did not get any staining with the control Fc protein in the IAPE Env expressing cells and the control amphotropic MLV (Ampho) Env expressing ones. Thus, expression of the IAPE Env by a cell increases the specific binding of Ephrin A4. Finally, to definitely demonstrate a physical interaction between the 2 proteins, we performed a pull-down assay using the soluble recombinant version of the 2 proteins described above, together with control proteins (see scheme in Figure 3C): IAPE or syncytin1 His-tagged SU proteins were mixed with the soluble Ephrin A4-Fc protein (or controls) and incubated for 1 hour at 37°C. The Fc-tagged proteins were then pulled down using protein A-agarose beads, the beads were washed and the presence of associated SU proteins that may have co-precipitated was tested by Western blot analysis using an antibody directed against the His tag. As shown in Figure 3C, the IAPE SU protein was efficiently pulled down only when incubated with the Ephrin A4-Fc protein. The syncytin1 control SU protein was never recovered, indicating that the observed interaction is specific. This clearly shows that the IAPE Env and Ephrin A4 proteins physically interact. Altogether, these results demonstrate that the Ephrin A4 protein is a receptor for the IAPE Env.

### Other Ephrin A proteins can also be used as receptors by the IAPE Env

**Figure 4:**
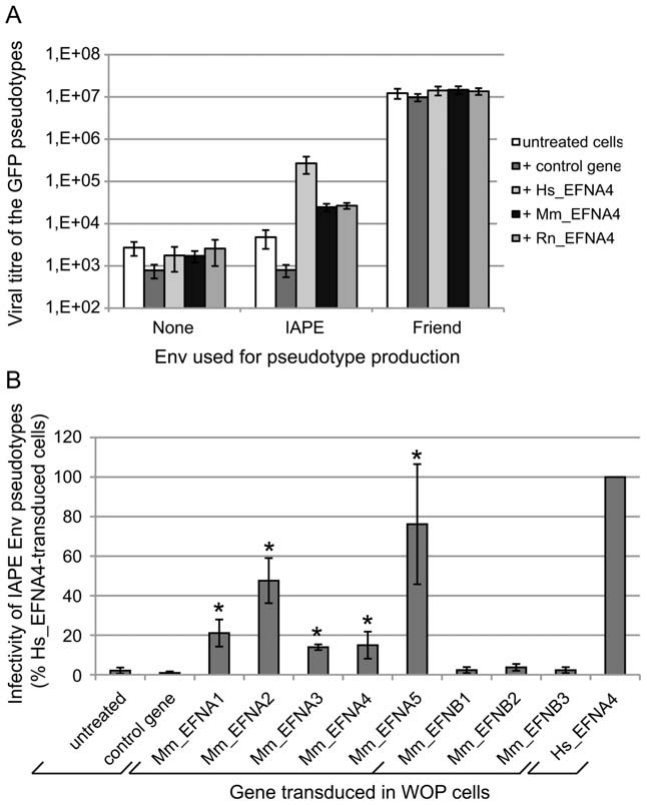
Characterisation of several Ephrin A proteins as receptors for the IAPE Env protein. (A) Comparison of the efficacy of human (Hs), mouse (Mm) and rat (Rn) Ephrin A4 proteins as receptors for IAPE Env pseudotypes. WOP cells were transduced with a lentiviral vector encoding one of the 3 versions of EFNA4, or a control gene. Three days later, they were challenged with GFP-marked lentiviral particles pseudotyped with the IAPE Env, the Friend ecotropic Env or no Env (none). Viral titres were measured by FACS three days post infection. The results (mean titre ± standard deviation) are from 5 independent experiments. (B) Test of the mouse Ephrin A and Ephrin B proteins for their activity as receptors for the IAPE Env pseudotypes. WOP cells were transduced with lentiviral vectors containing the different EFNA/B cDNA or a control gene, and tested for their ability to be infected with IAPE Env pseudotypes as described in (A). Results (mean ± standard deviation calculated from 3–4 independent experiments) are expressed as the percentage of the titre measured for each gene as compared to that obtained with the human EFNA4 (Hs_EFNA4, shown on the right). Asteriks indicate values significantly different (p<0.05) from that obtained with the untreated cells (unpaired Student test).

Since the cDNA library we used to identify the receptor of the IAPE Env was made from Huh7 cells, we tested de facto the human version of this gene for its receptor activity. However, IAPE elements are murine endogenous retroviruses and, in the course of our study, we could not identify a mouse cell line that was susceptible to infection by IAPE Env-pseudotyped viruses, which could have indicated the IAPE family was behaving as a xenotropic retrovirus. We thus decided to test the mouse version of Ephrin A4 for its activity as a receptor, as well as the rat one since it is another species containing IAPE elements. The cDNAs of these two genes (see Methods) were cloned into a lentiviral vector that we used to transduce mouse WOP cells before challenging them with IAPE Env pseudotypes, as described previously. The results of this experiment are shown in Figure 4A. As expected for cells not expressing a functional receptor, the presence of IAPE Env on the pseudotyped particles does not notably increase the titre as compared to the no-Env control pseudotypes (average fold increase in the untreated cells 1.6±0.5). As expected, the fold increase observed for cells transduced with a control gene (1.1±0.3) is not significantly different (unpaired Student test), unlike what is observed when the cells are transduced with the EFNA4 cDNAs (fold increases: 231±151, 16±7 and 16±10 for the human, mouse and rat EFNA4 genes, respectively; p<0,05 in all 3 cases). This indicates that the mouse and rat genes can be used as receptors by the IAPE Env, even though they are around ten times less efficient than the human version of Ephrin A4. We ensured both by qRT-PCR (not shown) and staining for Ephrin A proteins (Figure S1) that the expression levels of all constructs were similar and cannot account for the observed differences. To characterise further the interaction between the IAPE Env and Ephrin A4 proteins, we used the soluble His-tagged IAPE SU protein as a probe and stained WOP cells transduced with the different versions of the EFNA4 gene or a control. We were able to show that the lower receptor activity we observed with the rodent EFNA4 genes is linked to a marked decrease in their IAPE Env binding (Figure S1).

As mentioned earlier, Ephrin A4 is a member of a multigenic family of proteins, the Ephrin As, that are all related GPI-anchored membrane proteins. We thus decided to test all five members for their activity as a receptor for the IAPE Env, as well as the three EFNBs genes, which are also related, but encode integral membrane proteins (called Ephrin B proteins) (reviewed in [14], [15]). We cloned all 8 mouse cDNAs, introduced them in a lentiviral vector and tested them as previously described. Figure 4B shows the IAPE Env pseudotype titres we measured on the transduced WOP cells, expressed as percentages of the values obtained with the human version of EFNA4. As shown in the figure, none of the Ephrin B proteins can be used as a receptor by the IAPE Env, whereas all five Ephrin A members are functional (see legend for statistical analysis). However, three of them (Ephrin A1, 3 and 4) show only limited receptor activity, whereas Ephrin A2 and Ephrin A5 are nearly as efficient as the human Ephrin A4 protein. As before, we checked that these differences are not due to variations in the Ephrin expression levels (Figure S1 and qRT-PCR not shown). Finally, staining of the transduced cells with the soluble His-tagged IAPE SU protein (Figure S1) indicated that the mouse Ephrin A2 and Ephrin A5 proteins have a higher affinity for the IAPE Env compared to the other members of the family for which we could not detect any specific binding. This could account for the differences we observed in their receptor activity.

### Ephrin A expression pattern in vivo is compatible with the amplification of the IAPE family via re-infection of the germline

**Figure 5:**
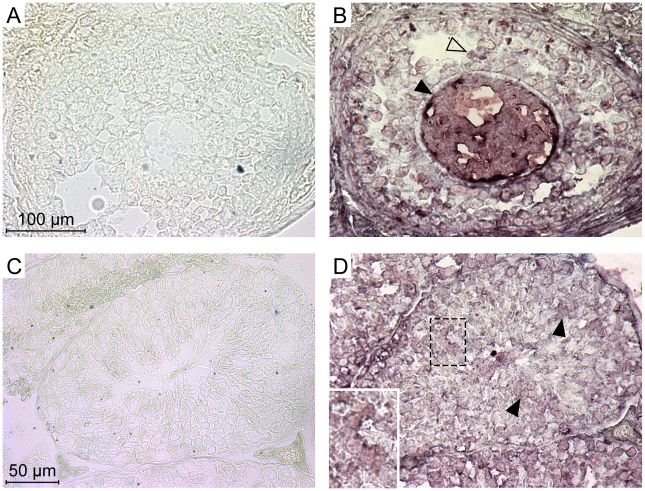
Expression of the Ephrin A proteins in mouse ovary and testis detected by immunohistochemistry. Ephrin A proteins in these serial cryosections were labelled using a commercial soluble EphA7-Fc tagged protein (a soluble form of EphA7 that can bind all-Ephrin A proteins) (B and D), or a Fc-only control protein (A and C). A and B show high magnifications of a Graäfian follicle with the oocyte (filled arrowheads) surrounded by follicle cells (open arrowheads). C and D show cross-sections of seminiferous tubules, with spermatozoa in the central lumen. Filled arrowheads point to stained germinal cells located apart from the periphery of the tubule (inset: higher magnification of stained cells).

The experiments above indicate that each of the five members of the Ephrin A family can be used as a receptor by the IAPE Env in ex vivo experiments. In a previous study, we demonstrated that the IAPE elements behave like infectious retroviruses, i.e. they produce extracellular particles that can infect cells, but they are unable to undergo intracellular retrotransposition cycles, unlike the related IAP elements [10]. The mouse genome contains around 200 copies of this family, indicating that it replicated quite successfully during rodent evolution. We thus tried to detect expression of the Ephrin A proteins in the germline of mice, since their presence should be necessary to account for the amplification of the IAPE elements through successive re-infections of the germline. First, we quantified the expression of the five EFNA genes in a panel of mouse organs using qRT-PCR. We could detect a strong expression of some of these genes in the embryos and the adult brain, as expected for genes involved in developmental processes and axon guidance. We could also detect some expression for EFNA1, 2, 4 and 5 in the adult ovary, and for EFNA2 and 3 in the testis (Figure S2). We therefore performed immunohistochemistry experiments on cryosections of these two organs in order to confirm these data, using as a probe a recombinant EphA7-Fc protein (a soluble form of EphA7 that can bind all-Ephrin A proteins, [14]), as previously described in ref. [19]. As shown in Figure 5, we could see a specific staining on both organs (panels A, B for the ovary, C, D for the testis), not obtained with the control “Fc-only” samples (panels A and C). In the ovary, a strong staining was seen in the oocytes, as well as to a lower extent in some cells of the growing follicles. In the testis, we saw also a specific staining of some cells within the seminiferous tubules. Some of the staining was quite a distance within the tube (panel D), in a location containing only germline cells since the somatic Sertoli cells are restricted to the periphery of the tubes. In both cases, the staining was strong and rather ubiquitous, which may be due to the fact that the probe can detect all Ephrin A proteins. We thus did a series of experiments using antibodies specific for Ephrin A2 or Ephrin A5, which are the most efficient murine receptors for IAPE Env. As shown in Figure S3, in the ovary each of these two antibodies gave a specific staining pattern, with Ephrin A2 being detected mostly in the oocytes and in the interstitial cells of the ovary stroma whereas Ephrin A5 was detected mostly in follicle cells, and at a lower level in the oocytes. This is consistant with the staining observed using the EphA7-Fc probe that stains all Ephrin A proteins, and confirms that efficient IAPE receptors are expressed in murine female germ cells. In the testis, the Ephrin A2 antibody gave a staining similar to that observed using EphA7-Fc, with spermatozoa stained as well as some dispersed cells inside the seminiferous tubules. The Ephrin A5 antibody gave a less intense signal, and was found mostly in spermatozoa. According to these data, the expression pattern of the Ephrin A proteins, found both in the oocyte and in male germline cells, is thus compatible with their role in the amplification of the IAPE family of endogenous retroviruses.

## Discussion

In this study, we set to identify the protein used by the endogenous retroviral family IAPE to enter its target cells. We used a well-established strategy aiming at complementing a refractory cell line with a cDNA library in order to identify genes able to render the cells sensitive to infection. With this method, we found that the human gene EFNA4 encodes a functional receptor, and that its mouse homolog, as well as the other members of the Ephrin A family, also function as receptors for IAPE. By using recombinant soluble proteins, we could further demonstrate a direct interaction between Ephrin A4 and IAPE Env, ruling out any artefact in the screening that could have led us to the identification of an inducer of the bona fide receptor gene.

**Figure 6:**
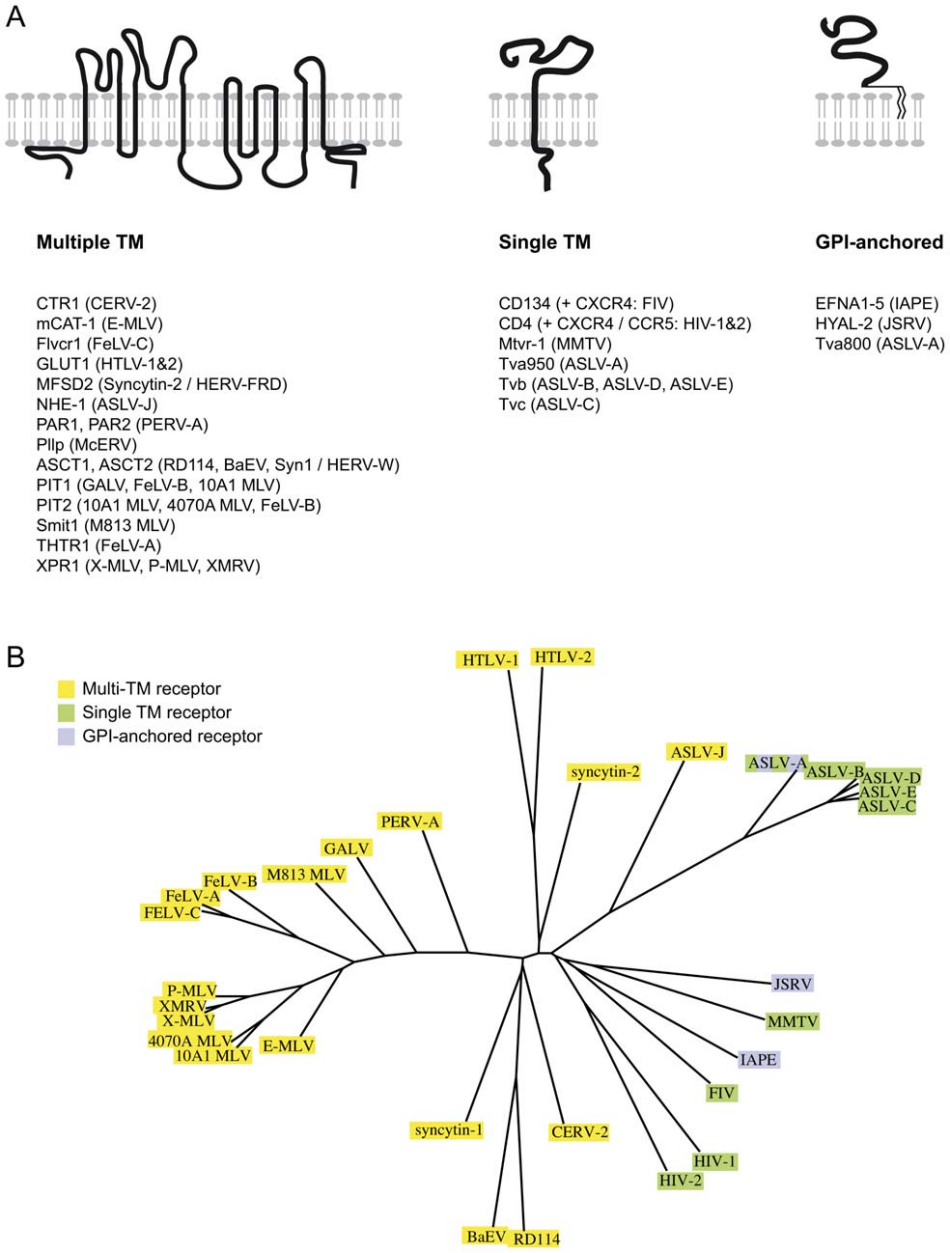
Classified retroviral receptors and dendrogram of the corresponding retroviral envelopes. A. Scheme of the three types of proteins that are used as receptors by various retroviruses. Examples of each type are provided below, with the name of the receptors indicated on the left followed by the name of the virus(es) using them in brackets. B. Dendrogram of the envelopes listed in A. Those using receptors with multiple membrane domains are in yellow, single membrane domain in green and GPI anchor in purple.

The Ephrin A proteins are all GPI-anchored membrane proteins that are used in vivo as ligands for the multigenic ephrinA receptor membrane proteins, and are involved in numerous biological processes ranging from axonal guiding to insulin regulation or immune processes (reviewed in [14], [15], [20]). Over the years, a series of cellular proteins used by retroviruses as receptors have been identified (reviewed in [21], [22] and see Figure 6A). In most cases, the encoded proteins have been shown to contain several transmembrane domains. In other cases, the receptor possesses a single transmembrane domain, like the transferrin receptor that is used by the Mouse Mammary Tumor Virus (MMTV) [23]. But few occurrences of GPI-anchored proteins used as receptors have been reported: there is hyaluronidase 2 (Hyal2), the receptor of Jaagsiekte Sheep RetroVirus (JSRV) [24], and one of the isoforms of TVA, the receptor used by the avian sarcoma and leukosis virus (the other isoform being a single transmembrane protein) [25]. The apparent oddity of the Ephrin A proteins as receptors, with their GPI-anchor, may mostly be due to the fact that the majority of the receptors identified so far are used by gammaretroviral and closely related envelope proteins. All of them share a common organisation (reviewed in [2], [26], [27]): their TM subunit is particularly conserved, especially around the so-called immunosuppressive domain (CKS17) that is followed by a CX_6–7_C(C) motif, and even if their SU subunits are less conserved, they still possess common features, including a CWLC (consensus CXXC) motif that is thought to be involved in SU-TM interaction through the TM CX_6–7_C(C) motif. This common structure may be a reason why they all recognise a same class of proteins, containing multiple membrane spanning domains (Figure 6A). IAPE or JSRV Env belong to a far less described group of retroviral envelope proteins, whose only features shared with the gammaretroviral group are a furin SU-TM cleavage site (R,X,R/K,R) and the TM CX_6–7_C(C) motif [26]. No similarity between the 2 groups can be detected within the SU subunit. It is therefore likely that this second group of envelope proteins may have evolved to make use of a different subset of receptors (Figure 6B).

Another unusual feature of the IAPE envelope we uncovered in this study is its apparent loose recognition specificity, since all 5 mouse Ephrin A proteins can be used as a receptor. These proteins are all related, but they have evolved independently for millions of years, which has resulted in a significant divergence (identity rate between 62 and 68% in amino acids). Accordingly, considering this rather low conservation, it was unexpected to observe that the five proteins are all functional receptors. However, this level of conservation is probably enough to maintain common structural features enabling recognition of all five proteins by a same molecule. This is supported by the natural ligands of the Ephrin A proteins, the EphA receptors, which show a broad specificity too: most of them can recognise all or most of the Ephrin A proteins, even though the affinity for one or the other Ephrin A can vary by more than one log as measured in ex vivo assays (reviewed in ref. [14]). It is thus not so surprising to have the IAPE envelope showing the same sort of general family-wide recognition. It is also in agreement with the observation that the Nipah and Hendrah viruses can use both Ephrin B2 and Ephrin B3 proteins as their receptor, even if Ephrin B2 is preferred [28], [29]. Interestingly we could not detect any similarity between the IAPE SU domain of the envelope (responsible for the recognition of the receptor) and the EphA proteins, suggesting that the ability to interact with the Ephrin A proteins was a de novo acquisition by the IAPE retroviral elements, and not the result of a recombination with a cellular copy of an EphA gene.

On an evolutionary point of view, the IAPE use of the 5 Ephrin A proteins as receptors is interesting considering its endogenous status. Until now among the reported cases, only one or sometimes two different proteins can be used as a receptor by a given retroviral Env (reviewed in [21], [22]). With all Ephrin A proteins being functional receptors, the IAPE family seems rather unusual. However, it could be hypothesised that this feature has been a great asset for the amplification and survival of the family. First because of the very nature of the EFNA genes, which are involved in a series of developmental processes and are widely expressed (reviewed in [14], [20] and data in Figure 5 and Figures S2 and S3), particularly very early in the development, when the germline is still readily accessible to viral particles. This high expression combined with the ability to infect through any of the five Ephrin A proteins suggests that the number of possible target cells is quite high. Second because, with these five proteins being functional receptors, it seems virtually impossible for the host to evolve so as to be protected against re-infection of its germline by IAPE viral particles. Such an escape mechanism is a common theme in host-pathogen interactions, and indeed has occurred recently in the mouse lineage, with most modern laboratory mice being protected from their own xenotropic endogenous proviruses thanks to a recent point mutation in their unique xpr1 receptor gene, which renders it non-functional for xenotropic virus entry (reviewed in [30]). In the case of the IAPE family however, the inactivation of the five Ephrin A proteins as receptors while maintaining their physiological function is most certainly an impossible task, and this could explain why this family was so successful and is still maintained within the mouse genome concomitantly with its envelope-less strictly intracellular IAP progeny.

## Methods

### Plasmids

CMV-driven mammalian expression vectors for the IAPE Env (IAPE D2*) and the other envelope proteins used as controls have been described previously [10], [31], as well as the plasmids used to generate lentiviral HIV-1 pseudotypes [32]. New self-inactivating lentiviral vectors (pSIN) were derived from pHR'SIN-cPPT-SEW [33] by replacing the GFP open reading frame (BamHI – NotI fragment) by that of other genes: LacZ gene, hygromycin resistance gene, Cherry fluorescent protein encoding gene, cDNAs of EFNA and EFNB genes. The latter were obtained by RT-PCR performed on purified RNA extracted from mouse embryos (mouse genes) or the 208F cell line (rat EFNA4). The expression plasmids for the soluble Fc-tagged versions of Ephrin A4, EphA2 and the Fc-only control were described previously [18]. The vector used to produce the soluble His-tagged SU IAPE env subunit was generated by replacing the TM domain in the IAPE Env expression plasmid by a VHRGSH_6_ sequence placed just downstream from the cleavage site which was changed into an AAAR sequence. The control plasmid encoding the soluble His-tagged SU of Syncytin1 was generated as described in ref. [17]. All fragments generated by PCR were sequenced to ensure that no mutation had been introduced during this step.

### cDNA library

The cDNA library was custom-made by Invitrogen using mRNA extracted from Huh7 cells, which was reverse-transcribed and cloned in the pLenti6/V5-Dest lentiviral vector.

### Quantitative RT-PCR

Total RNAs were extracted using the RNeasy extraction kit (Qiagen) and treated with DNase I (Ambion). 1 µg was used for each RT reaction using the MLV reverse-transcriptase (Applied Biosystems). Quantitative PCR was done using 5 µL of a 1/25 dilution of the cDNAs in a final volume of 25 µL by using SYBR Green PCR Master Mix (Applied Biosystems). PCR was carried out using an ABI PRISM 7000 sequence detection system. The efficacy of the PCR reaction was checked for each primer pair using serial dilutions of a reference sample and found to be more than 90%. The transcript levels were normalized relative to the amount of RPLO transcripts using the ΔΔCt method. Samples were assayed in duplicate.

### Cell culture, transfection, virus production and infection assays

293T, WOP and Vero cells were grown at 37°C and 5% CO_2_ in Dulbecco's modified Eagle's medium (DMEM) supplemented with 10% heat-inactivated foetal calf serum (Invitrogen), 100 µg/mL streptomycin, and 100 U/mL penicillin. Hygromycin selection was performed for two weeks using 400 u/mL hygromicin B (Calbiochem). For transfection, 293T cells were seeded at approximately 20% confluence. The day after seeding, they were transfected using Fugene 6 (Roche) or JetPrime (Polyplus transfection) following the manufacturer instructions, except we used 3 µg total DNA per 6 cm dish (this quantity was adapted proportionally to the dish surface when transfections were performed in different scales). The cells were washed and placed in fresh medium the day after transfection. For virus production, we used the following ratio for the plasmids: 8.91 (HIV Gag-Pol expression vector): 1; pSIN lentiviral vector: 1.5; Env expression vector: 0.3. The viral particles-containing supernatants were collected at day 3 post transfection, and passed through 0.45 µm filters before use. Infections were performed by adding viral supernatants to target cells in the presence of polybrene (8 µg/mL), and complemented with cyclosporine A (5 µM) in the case of Vero cells to abrogate TRIM5α–mediated restriction of the HIV1-derived particles [34]. In some experiments, the infection rates were increased by subjecting the cells to spinoculation (centrifugation for 2h30 at 1200 g, 25°C) just after adding the viral supernatants (this step also slightly increased the background infection level measured with the No Env Pseudotypes). Infection was detected three days post infection by staining the cells with X-Gal for the LacZ reporter gene, or by FACS for the GFP reporter gene. In the latter case, we used samples containing 5–15% GFP positive cells and calculated a viral titre from the volume of supernatant used for the infection and the number of target cells that were seeded. The 5–15% window was chosen to ensure we were still in the linear zone of the infection curve, where positive cells have only been infected by one particle.

### Cell staining and FACS analysis

For the cell staining experiments, we used Fc-tagged soluble recombinant proteins (Ephrin A4-Fc, EphA2-Fc and the control Fc-only, described in [18]) and His-tagged soluble Env SU (IAPE-His and Syncytin1-His) as “probes”. These were produced by 293T cells in a serum-free medium (OptiMEM, Invitrogen) after transient transfection with a CMV-driven mammalian expression vector using Fugene6 (Roche). The protein-containing supernatants were collected at day 2 post transfection and passed through 0.45 µm filters before use. Samples were analysed by Western blot to ensure the proteins were produced at similar levels in the supernatants. The cells to be stained (10^6^ per sample) were detached using PBS 5 mM EDTA, washed in PBS and incubated for 1 h at 37°C in neat supernatant containing the recombinant protein, washed twice in PBS, 2% FCS, 0.1% sodium azide and incubated for 30 min with a fluorescent antibody (Alexa 488 Anti mouse IgG, Molecular Probes or Alexa 488 PentaHis antibody, Qiagen) at 4°C. They were then washed 3 times in PBS, 2% FCS, 0.1% sodium azide, resuspended in PBS, 0.1% sodium azide and fixed with paraformaldehyde before FACS analysis.

### Pull-down assay and Western blot

Fc-tagged and His-tagged soluble recombinant proteins were produced as described above. Protease inhibitors (cOmplete protease inhibitors cocktail tablets, Roche) were added to the supernatants containing the recombinant proteins. 700 µL of each supernatant (His-tagged SU and Fc-tagged protein) were mixed and incubated for 1 h at 37°C with gentle agitation. Then protein A-agarose beads (Pierce, 20 µL packed beads per sample) were washed twice in PBS and saturated in PBS, 0.5% BSA before they were added to each sample complemented with BSA (0.5% final concentration). After a 1 h incubation at 37°C with gentle agitation, the beads were washed 5 times in PBS, Tween 0.1% before 150 µL of Laemmli buffer containing 10% ß-Mercaptoethanol were added to each sample that was then boiled for 5 min.

For Western blot analyses, reduced samples (5 µL of neat supernatant or 8 µL of the pull-down assay product) denatured in Laemmli buffer or LDS loading buffer (Invitrogen) were subjected to SDS-PAGE using gradient precast gels (Novex 4–12% Bis-Tris gels, Invitrogen). After migration, proteins were transferred onto a nitrocellulose membrane using a semi dry transfer system. His-tagged proteins were detected using the Penta-His HRP antibody (Qiagen), and the Fc-tagged ones using the ECL sheep anti mouse IgG (HRP-linked F(ab′)2 fragment, Amersham). The antibody directed against the IAPE Env has been previously described [10], and the antibody used to detect the amphotropic MLV Env was a goat anti Rauscher Leukemia Virus gp70 [originally obtained from the National Cancer Institute, Frederick, MD].

### Immunohistochemistry experiments

8–9 week old C57Bl/6 mice were used. The testis and ovaries were fixed by immersion in 4% paraformaldehyde in 0.1 M sodium phosphate pH 7 for 5 h, followed by 20% sucrose in PBS overnight. The tissues were embedded in Tissue-Tek OCT (Sakura) and sections were cut using a cryostat. Staining of total Ephrin A proteins was performed using the EphA7-Fc fusion protein (stains all 5 Ephrin A proteins, see [14]) or its control Fc-only protein (10–25 µg/mL, both purchased from R&D Systems) essentially as described in ref. [19], except that the blocking was performed in the presence of rat anti mouse CD16/CD32 used at a 1/50 dilution (Pharmingen). Staining of specific Ephrin A proteins was done using either a goat anti mouse Ephrin A2 antibody (R&D systems) or a rabbit anti Ephrin A5 antibody (Novus Biologicals), following the recommendations provided. Revelation was done using Alkaline Phosphatase-linked secondary antibodies (donkey anti-Rabbit IgG and bovine anti-Goat IgG for Ephrin A2 and Ephrin A5, respectively, Jackson ImmunoResearch) and NBT/BCIP substrate (1-Step NBT/BCIP plus Suppressor, Thermo Scientific).

### Accession numbers

The sequences we used as references for the cloned EFNA and EFNB genes are as follows: Hs_EFNA4: NM005227; Rn_ENFA4: NM001107692, Mm_EFNA1: NM010107, Mm_EFNA2: NM007909, Mm_EFNA3: NM010108, Mm_EFNA4: NM007910, Mm_EFNA5: NM207654, Mm_EFNB1: NM010110, Mm_EFNB2: NM010111, Mm_EFNB3: NM007911.

IAPE-D2* Env sequence can be deduced from the IAPE D2 provirus [10]: AC131339, pos: 143356–35028. Similar results were obtained using IAPE-D1 Env whose sequence can be deduced from AC123738, pos: 161181–152862.

### Ethics statement

This study was carried out in strict accordance with the French and European laws and regulations regarding Animal Experimentation (Directive 86/609/EEC regarding the protection of animals used for experimental and other scientific purposes). The protocol was approved by the Institut Gustave Roussy Animal Experiment Committee (MENRT n° 26).

## Supporting Information

Figure S1 Characterisation of WOP cells transduced with the different EFNA and EFNB genes tested in this study. The WOP cells transduced with the series of EFNA/EFNB cDNA-containing LVs and tested for their ability to be infected by IAPE Env pseudotypes (see Figure 4) were stained with different soluble proteins and subjected to FACS analysis. Staining with the EphA2-Fc protein (in green) indicates the level of Ephrin A protein expression. Cells transduced with EFNAs (Hs_EFNA4, Rn_EFNA4 or each of the 5 mouse EFNAs) show a strong increase in EphA2-Fc staining, indicating that the transduced EFNA cDNAs are all highly and equally expressed. There is also some EphA2-Fc staining observed with the EFNB1 and EFNB2 transduced cells (but much weaker), indicating that EphA2 can to some extent cross-label these Ephrin B proteins. Staining with the IAPE SU-His protein (red) was used to test whether the different Ephrin A and B proteins can interact with IAPE Env. The 3 “best” EFNA cDNA that can render WOP cells infectable by IAPE Env pseudotypes (Hs_EFNA4, Mm_EFNA2 and Mm_EFNA5) are the only ones that can bind the IAPE SU-His protein, suggesting they have a stronger affinity for the IAPE envelope than the other EFNA genes tested. The control protein sample shown in the figure (filled in light grey) corresponds to cells stained with a control His-tagged soluble protein and an anti-His Alexa 488 secondary antibody; staining with a Fc-only protein and the corresponding Alexa 488 secondary antibody gave the same profile (not shown).
(TIF)

Figure S2 Quantification of the EFNA genes expression in a panel of mouse organs. The RNA levels of the 5 mouse EFNA genes were measured in a panel of mouse tissues (from 8–9 week old C57Bl/6 mice, except for embryos and placentas that were aged 11.5 d) by quantitative RT-PCR. Reactions were performed essentially as described in the Methods section, except that in this case the transcript levels for each gene were measured using serial dilutions of a reference sample as an internal standard. The transcript levels in the different tissues were normalized relative to the amount of RPLO transcripts, and are expressed for each EFNA gene as percentage of the maximum expression detected.
(TIF)

Figure S3 Detection of the Ephrin A2 and Ephrin A5 proteins by immunohistochemistry. Expression in mouse ovary (A–D) and testis (E–H). Ephrin A2 (B and F) and Ephrin A5 (D and H) proteins in these serial cryosections were labelled using commercial antibodies specific for each protein or, as a negative control, using an irrelevant primary antibody that was generated in the same species and subjected to similar purification (control for Ephrin A2 staining, A and E)) or the secondary antibody only (control for Ephrin A5 staining, C and G). Abbreviations: oocyte: Oo, follicular cells: FC, interstitial cells: IC, spermatozoa: Sp.
(TIF)
