# Supplementary figures and images for: The Mouse IAPE Endogenous Retrovirus Can Infect Cells through Any of the Five GPI-Anchored EphrinA Proteins
Source: PLoS Pathog. 2011 Oct 20;7(10):e1002309. doi: 10.1371/journal.ppat.1002309 (PMC3197615; doi:10.1371/journal.ppat.1002309)

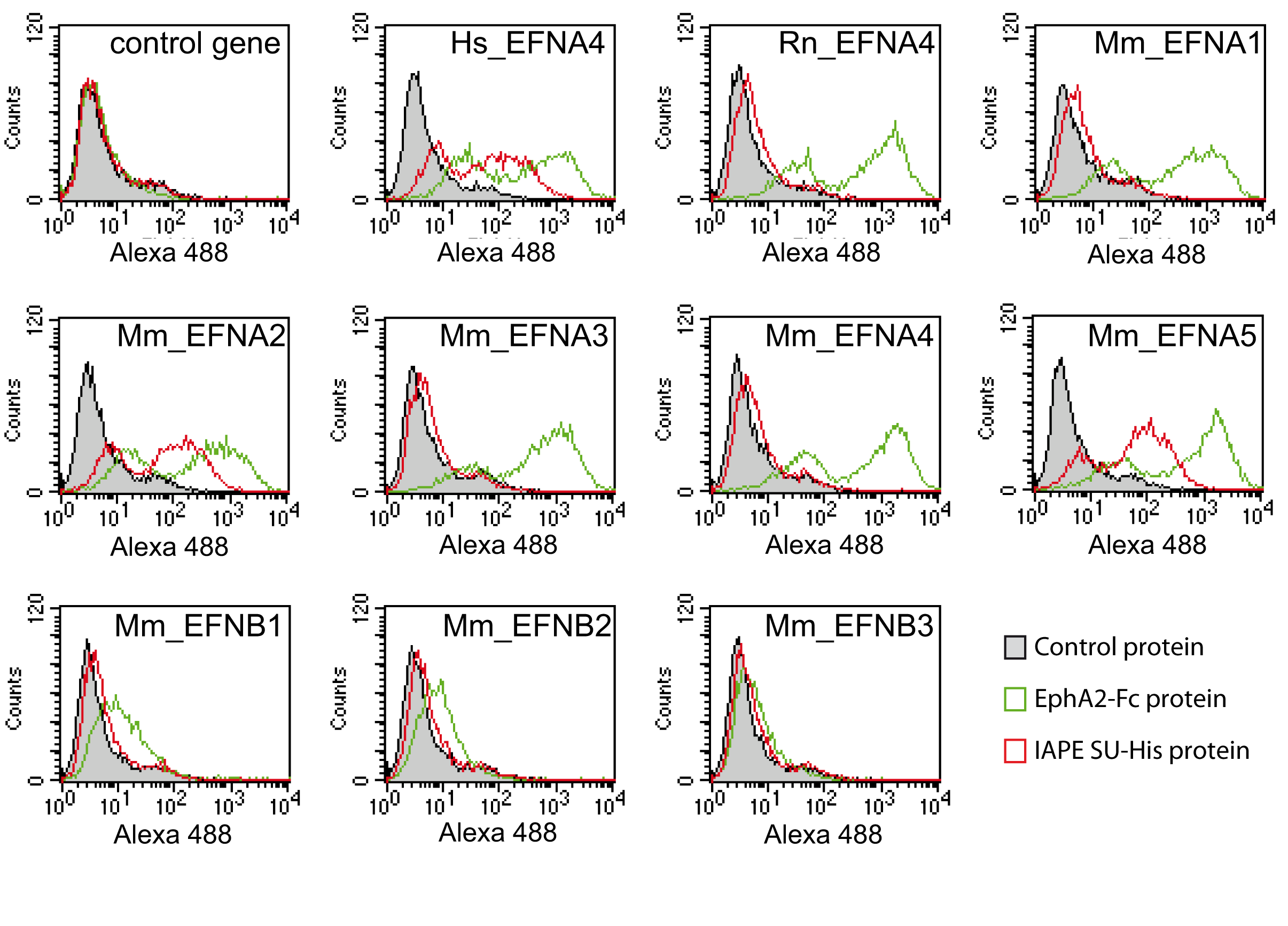

Supplement: Figure S1 — Characterisation of WOP cells transduced with the different EFNA and EFNB genes tested in this study. The WOP cells transduced with the series of EFNA/EFNB cDNA-containing LVs and tested for their ability to be infected by IAPE Env pseudotypes (see Figure 4) were stained with different soluble proteins and subjected to FACS analysis. Staining with the EphA2-Fc protein (in green) indicates the level of Ephrin A protein expression. Cells transduced with EFNAs (Hs_EFNA4, Rn_EFNA4 or each of the 5 mouse EFNAs) show a strong increase in EphA2-Fc staining, indicating that the transduced EFNA cDNAs are all highly and equally expressed. There is also some EphA2-Fc staining observed with the EFNB1 and EFNB2 transduced cells (but much weaker), indicating that EphA2 can to some extent cross-label these Ephrin B proteins. Staining with the IAPE SU-His protein (red) was used to test whether the different Ephrin A and B proteins can interact with IAPE Env. The 3 “best” EFNA cDNA that can render WOP cells infectable by IAPE Env pseudotypes (Hs_EFNA4, Mm_EFNA2 and Mm_EFNA5) are the only ones that can bind the IAPE SU-His protein, suggesting they have a stronger affinity for the IAPE envelope than the other EFNA genes tested. The control protein sample shown in the figure (filled in light grey) corresponds to cells stained with a control His-tagged soluble protein and an anti-His Alexa 488 secondary antibody; staining with a Fc-only protein and the corresponding Alexa 488 secondary antibody gave the same profile (not shown). (TIF) [file ppat.1002309.s001.tif]

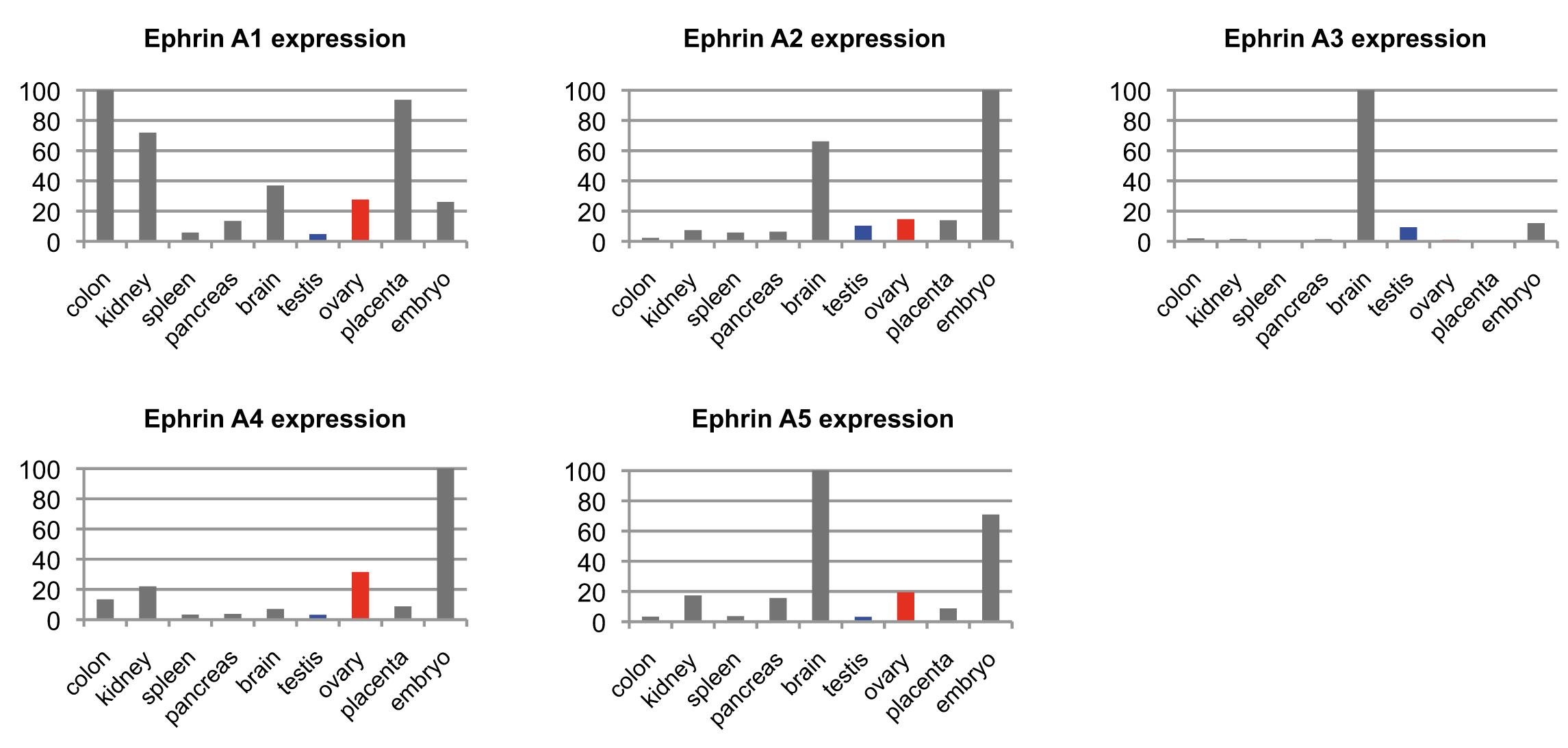

Supplement: Figure S2 — Quantification of the EFNA genes expression in a panel of mouse organs. The RNA levels of the 5 mouse EFNA genes were measured in a panel of mouse tissues (from 8–9 week old C57Bl/6 mice, except for embryos and placentas that were aged 11.5 d) by quantitative RT-PCR. Reactions were performed essentially as described in the Methods section, except that in this case the transcript levels for each gene were measured using serial dilutions of a reference sample as an internal standard. The transcript levels in the different tissues were normalized relative to the amount of RPLO transcripts, and are expressed for each EFNA gene as percentage of the maximum expression detected. (TIF) [file ppat.1002309.s002.tif]

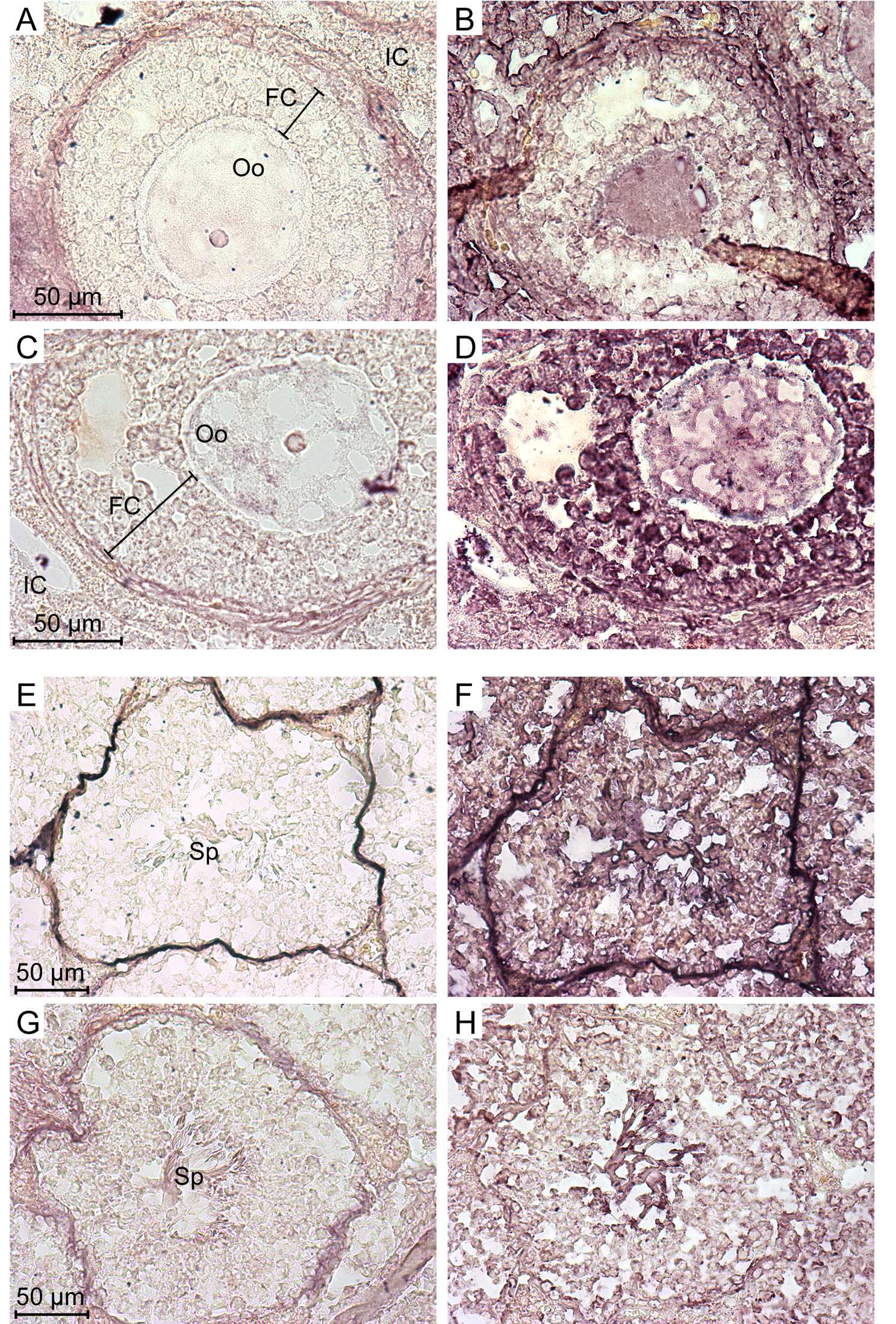

Supplement: Figure S3 — Detection of the Ephrin A2 and Ephrin A5 proteins by immunohistochemistry. Expression in mouse ovary (A–D) and testis (E–H). Ephrin A2 (B and F) and Ephrin A5 (D and H) proteins in these serial cryosections were labelled using commercial antibodies specific for each protein or, as a negative control, using an irrelevant primary antibody that was generated in the same species and subjected to similar purification (control for Ephrin A2 staining, A and E)) or the secondary antibody only (control for Ephrin A5 staining, C and G). Abbreviations: oocyte: Oo, follicular cells: FC, interstitial cells: IC, spermatozoa: Sp. (TIF) [file ppat.1002309.s003.tif]
